# Editorial: The impact of COVID-19 on social inequalities in sport and physical activity

**DOI:** 10.3389/fspor.2023.1308924

**Published:** 2023-10-19

**Authors:** Jim Lusted, Jacco Van Sterkenburg, Akilah Carter-Francique

**Affiliations:** ^1^ School of Education, Childhood, Youth and Sport, The Open University, Milton Keynes, United Kingdom; ^2^ Department of Media and Communication, Erasmus University Rotterdam, Rotterdam, Netherlands; ^3^ School of Education, Health, and Human Services, Benedict College, Columbia, MO, United States

**Keywords:** pandemic (COVID-19), sport, physical activity, social inequalities, discrimination

**Editorial on the Research Topic** The impact of COVID-19 on social inequalities in sport and physical activity

Living through the coronavirus, or COVID-19, pandemic fundamentally changed the way we view our lives and this legacy has been experienced in numerous ways. During the pandemic, it felt as if more people became aware of the importance of being physically active—to improve their health and help reduce the risks of the disease but also to manage the mental health challenges that COVID-19 restrictions caused. Interestingly, the importance of being physically fit and active as a prevention measure against COVID-19 was increasingly underlined by media and scholars alike (1). At the same time, government enforced lockdowns required many sport and leisure facilities and clubs to close their services, which meant opportunities to access formal physical activity decreased dramatically. Specific groups were especially framed as vulnerable to the impact of COVID-19, most notably the elderly, the chronically ill and disabled, and some ethnic minority groups (2, 3).

A variety of research studies indicate that people from marginalized groups, often living in deprived urban areas, were not only more vulnerable to the effects of COVID-19 but were more likely to be impacted negatively by the economic and social effects/realities of the pandemic (2, 4). Such groups were already confronted with a multitude of structural inequalities leading to poor health, income precarity and inadequate housing. Many of these groups were disproportionally affected during the pandemic, often being in “front-line” jobs such as healthcare or transportation, placing them at significantly higher risk of catching COVID-19 and unable to adapt their employment to work from home (unlike many others). Further, the insufficiency of government social support and/or safety nets were dramatically exposed during this time. Subsequently, these identified groups and others from disadvantaged social positions faced overlapping problems to include long-term inequalities and issues triggered by the changing social context created by the pandemic.

This disproportionate impact of the global pandemic consequently reinforced already existing social inequalities in society along the lines of race, ethnicity, nationality, disability, social class, age—and the intersections between all these factors (5). With this in mind, one should not be surprised to see existing barriers to inclusion and equality in sport and physical activity exacerbated by the social, economic and cultural impacts felt during and after the pandemic. The impact of the COVID-19 pandemic on participation in sport and physical activity amongst these and other groups has been profound—and evidence suggests that such groups have been slower to return to sport and physical activity since the pandemic (6).

The goal of the collection of articles in this Research Topic is to bring together research that explores in detail the impact of the COVID-19 pandemic on the nature and extent of the social inequalities that exist in sport and physical activity settings. Such research is important because it can identify specific issues, barriers and problems that can better inform the management strategies and programming efforts of sport organisations and practitioners in encouraging the return to, and new forms of, sport and physical activity for social groups who have been particularly affected by the specific social conditions created by the pandemic.

The articles that make up this exciting collection cover an impressively wide range of contexts and topics related to the impact of COVID-19 on inequalities in sport and physical activity today. Spanning three separate continents, the national contexts under consideration include Chile, South Korea, the Netherlands, and the Republic of Ireland. These significantly under-researched settings offer important insights into social inequalities that build upon research from more commonly explored regions such as the United States of America (USA), Canada, the United Kingdom, Australia, and New Zealand. For example, research in the USA examined a range of detriments, challenges and technological innovations that enhanced sport and physical activity engagement during and after the onset of COVID-19. Findings from scholars revealed the pandemic's impact on the physical and mental health of all groups but amplified how marginalized populations at the intersection of gender, ability, religion, and age continue to experience access and treatment discrimination. More specifically, Sport Based Youth Development research in the USA demonstrated how race and the Black Lives Matter Civil Rights Movement influenced sport & physical activity, participation, and organizational change (7).

The articles of this collection also offer a variety of angles from which to consider our Research Topic—exploring issues related to disability and sport participation; socio-economic status and neighbourhood in sport provision; access to sport and physical activity opportunities for migrant workers; and, the structure and support available for women's sport.

In their discussion of the impact of COVID-19 on participation in sport, Henriquez et al. (this collection) took a intersectional deep dive into a large-scale national survey of physical activity in Chile among disabled people, which covered the time of the COVID-19 pandemic. Their findings show that only 11.9% of disabled people met physical activity recommendations, while it was younger men with visual or hearing disabilities from the wealthiest economic status and with residency in central and southern Chile who were most likely to meet adequate levels of Physical activity.

Moving to The Netherlands, Hoekman et al. (this collection) explored the demographic trends in drop-out from sports club membership during the COVID-19 pandemic. Using neighbourhood characteristics such a socio-economic status, they found that club drop-out was less frequent in areas of higher socio-economic status containing an abundance of sports facilities. They discuss the implications of this situation for policy makers seeking to aid the return to sport and club membership for their populations.

Na and Park (this collection) take our attention towards South Korea and specifically the experiences of foreign workers in accessing sport and physical activity during the COVID-19 pandemic. They explore both the inhibiting and enabling factors that limited this population's access to sport and physical activity while also opening up new and different opportunities for foreign workers to remain active during the pandemic.

Finally, Crosson and Chumhaill (this collection) explore the impact of the COVID-19 on women's sport in the Republic of Ireland. They conceptualise the pandemic times as an “exposing force” that highlighted the widespread inequitable structures of sport in Ireland that continues to disadvantage women's participation—both in terms of the support available for clubs including funding for women's sport systems but also in the wider media coverage that women's sport receives.

Taken together, this collection of thought provoking articles offers an invaluable insight into the ways in which the COVID-19 pandemic has contributed to the ongoing social inequalities that exist in sport and physical activity. They have important implications particularly for those tasked with finding ways to build sport and physical activity participation through post-pandemic times.
